# Distinction of subtype-specific antibodies against European porcine influenza viruses by indirect ELISA based on recombinant hemagglutinin protein fragment-1

**DOI:** 10.1186/1743-422X-10-246

**Published:** 2013-07-30

**Authors:** Na Zhao, Elke Lange, Sybille Kubald, Christian Grund, Martin Beer, Timm C Harder

**Affiliations:** 1 Institute of Diagnostic Virology, Friedrich-Loeffler-Institut, Suedufer 10, Greifswald 17493, Germany; 2 Department of Experimental Animal Facilities and Biorisk Management, Friedrich-Loeffler-Institut, Suedufer 10, Greifswald 17493, Germany; 3 Institute of Immunology, Friedrich-Loeffler-Institut, Suedufer 10, Greifswald 17493, Germany

**Keywords:** Pandemic influenza, Swine, Mixing vessel, Subtype specificity, Sero-diagnostics

## Abstract

**Background:**

Serological investigations of swine influenza virus infections and epidemiological conclusions thereof are challenging due to the complex and regionally variable pattern of co-circulating viral subtypes and lineages and varying vaccination regimes. Detection of subtype-specific antibodies currently depends on hemagglutination inhibition (HI) assays which are difficult to standardize and unsuitable for large scale investigations.

**Methods:**

The nucleocapsid protein (NP) and HA1 fragments of the hemagglutinin protein (HA) of five different lineages (H1N1av, H1N1pdm, H1pdmN2, H1N2, H3N2) of swine influenza viruses were bacterially expressed and used as diagnostic antigens in indirect ELISA.

**Results:**

Proteins were co-translationally mono-biotinylated and refolded *in vitro* into an antigenically authentic conformation. Western blotting and indirect ELISA revealed highly subtype-specific antigenic characteristics of the recombinant HA1 proteins although some cross reactivity especially among antigens of the H1 subtype were evident. Discrimination of antibodies directed against four swine influenza virus subtypes co-circulating in Germany was feasible using the indirect ELISA format.

**Conclusions:**

Bacterially expressed recombinant NP and HA1 swine influenza virus proteins served as antigens in indirect ELISAs and provided an alternative to commercial blocking NP ELISA and HI assays concerning generic (NP-specific) and HA subtype-specific sero-diagnostics, respectively, on a herd basis.

## Background

Swine influenza is an economically important disease of pigs caused by infections with influenza A viruses (IAV). Economic losses are caused by retarded growth of fattening pigs due to influenza-induced or -aggravated respiratory disease. In addition, febrile influenza virus infections in sows may cause fertility problems [1]. Influenza viruses are an important factor in the polymicrobial respiratory syndrome of swine [2]. In contrast to human influenza, infections in swine do not appear to be seasonal, and virus circulation, especially in larger herds, is observed year-round [3]. Control of swine influenza is difficult and requires strict zoosanitary measures and herd vaccination programs. Licensed inactivated whole virus vaccines are available but high and continuous vaccine coverage within herds is required [4].

In addition to being affected by disease, pigs are considered to be an integral part of the wider epidemiology of influenza, bridging the avian influenza world to mammalian influenza. The porcine respiratory epithelium is lined by cells which express the two sialic acid glycan receptor structures to which avian- or mammalian-adapted IAV bind [5]. As such, pigs can be infected by IAV of human and of avian origin and this provides opportunities for reassortment between these viruses [6,7]. Following historic transspecies transmission events of IAV from human or avian sources to pigs some of these viruses have established stable circulating lineages in swine populations worldwide. These porcine lineages continue to reassort amongst each other and with other IAV of more recent human or avian origin. In Europe, this scenario has lead to current presence of at least four distinct lineages of swine influenza viruses in pig populations [8,9].

Since the late 1970s IAV of subtype H1N1 of purely avian origin (H1N1av) dominate the influenza epidemiology in swine in many European countries including Germany. This lineage, referred to as H1N1av, has fully adapted to swine and can be distinguished genetically and antigenically from current avian-adapted H1N1 viruses. Viruses of this lineage have sporadically been detected also in humans and turkeys in Europe due to single transmission events from infected pigs [10,11]. A second porcine lineage consists of viruses of subtype H3N2. The progenitor of the currently circulating porcine H3N2 strains originated from human-adapted H3N2 viruses which had caused the Hong Kong flu pandemic in 1968 [8]. In the early 1980s the descendants of this virus reassorted with H1N1av and, apart from the hemagglutinin H3 and the neuraminidase N2 segments, all further six genome segments were replaced with those of H1N1av [8]. In the early 1990s, a new porcine triple reassortant virus, H1N2, arose from reassortment events between human seasonal H1N1 and H3N2 and porcine H1N1av viruses. This porcine H1N2 virus carried hemagglutinin (HA) and neuraminidase (NA) of human origin and the cassette of six further segments of H1N1av [8]. These three lineages continue to co-circulate at varying prevalences in different European countries.

In 2009, a new human pandemic H1N1 strain (H1N1pdm) emerged. This virus carried reassorted gene segments from several American and Eurasian swine influenza lineages and was rapidly introduced from the human population to pigs [8]. Pigs proved to be highly susceptible to this virus and stable transmission chains were easily maintained [12]. To date H1N1pdm appears to circulate independently from the human population in swine in several countries worldwide. Recently we and others found evidence for the emergence of reassortants between H1N1pdm and authentic porcine influenza virus lineages in Germany. In particular, a reassortant lineage of subtype H1pdmN2 which carried seven segments of the H1N1pdm virus and a neuraminidase of subtype 2 that was derived from different porcine or human HxN2 lineages, circulated stably [9,13].

Measures aiming at control of swine influenza must be based on subtype-specific virological and serological diagnosis. Real-time RT-PCR (RT-qPCR) has provided ample applications for rapid and sensitive molecular virological diagnosis [14]. Serology in swine influenza has been found useful for retrospective epidemiological investigations e.g., [15], estimation of disease incidence e.g., [16], and control of vaccination success e.g., [17]. Several commercial ELISAs for detection of generic IAV nucleocapsid protein (NP)-specific antibodies in pigs are available and recommended for use e.g., [18]. With regard to detection of antibodies at the subtype-specific level the hemagglutination inhibition assay (HI) is still held gold standard despite several draw-backs of this method including time- and labour-consuming performance and dependence on labile and difficult-to-standardize components (viral antigen, erythrocytes). Furthermore, due to interference of antibodies against the viral neuraminidase component interpretation of HI-results are particularly difficult [19,20]. Antibodies to the IAV hemagglutinin protein (HA) become detectable by HI assay from the second week post infection on and HI titers correlate with protection from clinically overt disease [21,22].

Despite mentioned problems of HI assays very few swine influenza ELISA applications aiming at subtype differentiation at the antibody level have been reported and the current commercially available assays for subtypes H1 and H3 have not superseded HI assays, at least in Europe [23]. The reported lack of sensitivity of these assays may be related to the American origin of the IAV isolates used which are antigenically distinct from those circulating in Europe. Low specificity of these assays may be caused by use of whole virion preparations which contain group specific antigens such as the nucleocapsid protein.

Here we show that recombinant HA1 antigen of European swine influenza viruses which was bacterially expressed and refolded *in vitro* can be used in indirect ELISAs for detection and differentiation of subtype-specific antibodies in porcine sera.

## Results

### Bacterial expression of antigenic influenza HA1 protein

**Figure 1:**
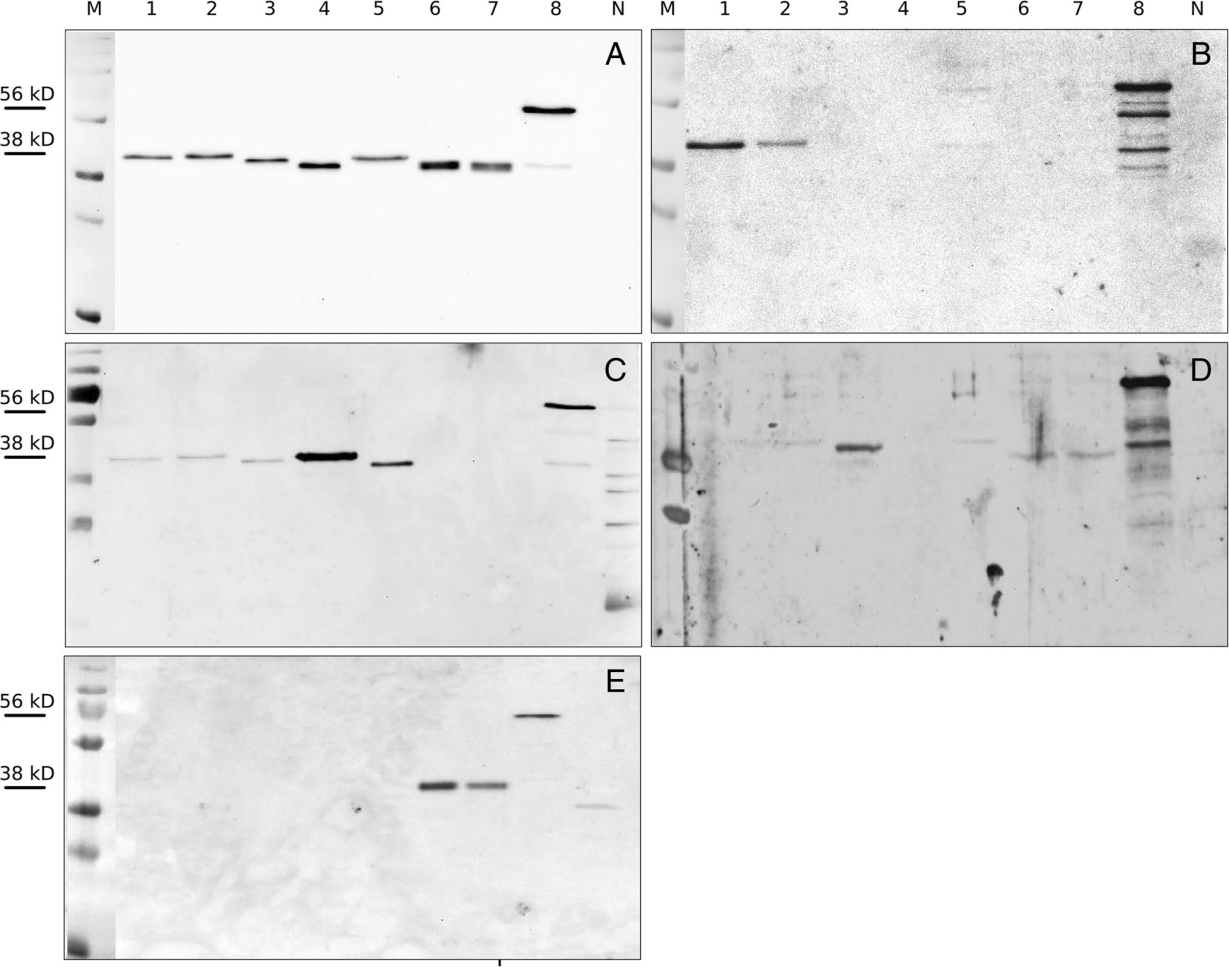
**Detection of biotinylated recombinant HA1 hemagglutinin and nucleocapsid proteins of European porcine influenza viruses.** An amount of approximately 20 μg of recombinant protein was loaded onto each lane and subsequently probed by an anti-biotin monoclonal antibody **(A)**, H1N1pdm (Bavaria/74) specific porcine serum OM46 **(B)** H1N1av (R681/11) specific ferret serum EL1 **(C)**, H1N2 (Bakum/1800)specific porcine serum OM1 **(D)** or H3N2 (R655/12) specific ferret serum EL2 **(E)** as described in materials and methods. Recombinant antigens in lanes: 1 – R26/11 H1N1pdm, 2 – R2035/11 H1pdmN2, 3 – R1207/10 H1N2, 4 – R1738/11 H1N1av, 5 – R1931/11 H1N2, 6 – R76/11 H3N2, 7 – R96/11 H3N2, 8 - nucleocapsid protein of A/swine/Germany/R96/2011 (H3N2), N - Bacterial lysate of Rosettagami cells carrying the *BirA* plasmid and an empty pET19b vector. The approximate molecular weight of recombinant HA1 (38 kD) and NP (56 kD) is indicated.

The HA1 protein fragments of seven recent swine influenza virus isolates (Table 1) were bacterially expressed (pET19b expression vectors) and co-translationally monobiotinylated by overexpressed bacterial *BirA* biotin ligase (pBIRAcm vector). In addition, the full-length nucleocapsid protein of one of the seven isolates was expressed similarly. The recombinant proteins sequestered into bacterial inclusion bodies. Purified bacterial inclusion body proteins were subjected to SDS-PAGE under reducing conditions for detection by Western blot analysis (Figure 1). Using an anti-biotin monoclonal antibody, recombinant proteins of expected molecular weights (HA-1 38 +/− 3 kD; NP ca, 56 kD) are depicted in Figure 1A. No further protein bands were identified and no biotinylated proteins were detected in a control which consisted of a clarified lysate of Rosettagami *E. coli* cells which had been co-transformed by an empty pET19b expression vector and pBIRAcm. The NP protein showed liability to proteolytical degradation as shown by a few and weak bands of lower molecular weight (Figure 1A, lane 8). Thus, the chosen bacterial co-expression system specifically produced biotinylated recombinant HA1 and NP proteins which could be successfully purified from inclusion bodies.

**Table 1:** Origin and properties of porcine influenza viruses used in this study for generation of recombinant proteins

**Identification**	**Subtype**	**HA^a^ Lineage**	**Sequence HA**	**Sequence NP**^**b**^
A/Germany/R26/2011	H1N1pdm	Pandemic 2009	EPI356430^c^	n.d.
A/swine/Germany/R1738/2010	H1N1	Eurasian avian-like	EPI411955	EPI426141
A/swine/Germany/R1931/2011	H1N2	Eurasian avian-like reassortant	EPI412039	n.d.
A/swine/Germany/R1207/2010	H1N2	Eurasian human-like reassortant	EPI411941	n.d.
A/swine/Germany/R2035/2011	H1pdmN2	Pandemic 2009 reassortant	EPI356453	n.d.
A/swine/Germany/R96/2011	H3N2	Eurasian human-like reassortant	EPI411978	n.d.
A/swine/Germany/R76/2011	H3N2	Eurasian human-like reassortant	EPI411965	n.d.

^a^ HA - hemagglutinin.

^b^ NP – nucleocapsid.

^c^ Sequence accession number in EpiFlu database.

n.d. - not determined.

The antigens reacted also with sera from IAV infected pigs and ferrets (Figure 1B-E; Table 2). The NP-antigen, although derived from a porcine H3N2 virus, was recognized by sera raised against four porcine IAV lineages (H1N1pdm, H1N1av, H1N2, and the homologous H3N2) as shown each in lanes 8 of Figure 1, panels B – D. A porcine serum raised against H1N1pdm was specific for the HA1 proteins of H1N1pdm and the reassortant H1pdmN2 (Figure 1B, lane 1 and 2). Serum from a ferret experimentally infected by an H1N1av isolate strongly reacted with homologous H1av HA1 proteins (Figure 1C, lane 4) but cross-reacted weakly also with other H1 HA1 recombinant proteins. An H1N2-specific porcine serum (Figure 1D) similarly showed strong specific staining with the homologous H1N2 HA1 (lane 3) and produced weaker signals with other recombinant HA1 antigens (e.g., lanes 2, 5). A ferret anti-H3 serum proved to be subtype-specific (Figure 1E, lanes 6 and 7).

**Table 2:** HI titres of porcine and ferret post infection sera used in Western blotting and indirect ELISA (homologous serum-antigen pairs depicted in bold)

**Serum raised against**	**Subtype**	**Host**	**Hemagglutination inhibiting titre**				
			**H1N1av**^**a**^	**H1N2**^**b**^	**H1N1pdm**^**c**^	**H1pdmN2**^**d**^	**H3N2**^**e**^
A/swine/Belzig/2/2001	H1N1av	Swine (OM8)	4*	1	2	1	1
A/swine/Germany/ R681/2011	H1N1av	Ferret (EL1)	**6**	1	6	2	2
A/swine/Bakum/1832/ 2000	H1N2	Swine (OM1)	1	**5**	1	1	1
A/swine/Bayern/74/ 2009	H1N1pdm	Swine (OM46)^f^	4	1	**6**	5	1
A/swine/Germany/ R2035/2011	H1pdmN2	Swine (OM15/4)	1	3	5	**6**	1
A/swine/Belzig/54/ 2001	H3N2	Swine (R4)	1	1	1	1	**6**
A/swine/Germany/ R655/2012	H3N2	Ferret (EL1)	1	1	2	2	**9**
Unknown, field serum	Pan	swine	6	6	5	5	6
Unknown, field serum	None	swine	1	1	1	1	1

Viruses that had been used for production of recombinant HA1 proteins were also used in HI assays:

^a^ – A/swine/Germany/R1738/2010.

^b^ – A/swine/Germany/R1207/2010.

^c^ – A/Germany/R26/2011.

^d^ – A/swine/Germany/R2035/2011.

^e^ – A/swine/Germany/R96/2011.

^* ^**–** Log_2_ titre (starting dilution 1:10).

### Determination of cut-off values for indirect ELISAs using recombinant NP and HA1 proteins

Using the biotin residue of the recombinant NP and HA1 proteins, streptavidin ELISA plates were coated with 0,5 μg of recombinant protein per well and then blocked. Sera to be tested were prediluted 1:200 and species-specific anti IgG antibodies conjugated with horseraddish peroxidase were used to detect bound antibodies in this indirect ELISA format. Seventy five sera from a total of 50 pigs were examined to determine cut-off values. These pigs originated from the influenza-negative minipig breeding cluster at FLI (n = 10) and from a swine farm which tested continuously seronegative against IAV. None of these sera tested positive in a commercial NP-specific blocking ELISA (ID.Vet, France). Mean extinctions were measured and standard deviations were calculated for the indirect ELISAs using recombinant NP and HA1 (Table 3). A porcine serum derived from a pig after multiple vaccination with four subtypes of European swine IAV possessed high HI titres against H1av, H1N2, H1pdm and H3N2 (Table 2) and was chosen as standard positive control. Likewise a negative porcine field serum with OD values close to the upper cut-off value of the set of negative sera was chosen as standard negative control. Based on these control sera (Table 3) S/P ratios were calculated for the set of 75 negative sera and the S/P mean plus 2 SD (recombinant HA1) or 3 SD (recombinant NP) were chosen as cut-offs in the examination of porcine field sera by indirect ELISAs. The exact cut-off values for each assay are presented in Table 3.

**Table 3:** Determination of cut-offs of indirect ELISAs based on recombinant NP and HA1 proteins

**Serum samples**	**Values**	**Recombinant antigens**					
		**NP**	**H1N1av**	**H1N2**	**N1N1pdm**	**H1pdmN2**	**H3N2**
Negative sera	Mean	0,11	0,03	0,06	0,03	0,04	0,14
N = 75	SD	0,08	0,03	0,10	0,08	0,07	0,14
	X + 2SD	0,35*	0,08	0,25	0,19	0,18	0,41
Positive standard	Mean	0,42	094	0,61	0,92	0,54	0,58
	SD	0,02	0,01	0,01	0,01	0,02	0,02
Negative standard	Mean	0,12	0,06	0,15	0,18	0,16	0,15
	SD	0,02	0,01	0,00	0,01	0,03	0,01

Viruses used for production of recombinant proteins:

NP, H1N1av – A/swine/Germany/R1738/2010.

H1N2 – A/swine/Germany/R1207/2010.

H1N1pdm – A/Germany/R26/2011.

H1pdmN2 – A/swine/Germany/R2035/2011.

H3N2 – A/swine/Germany/R96/2011.

* - For NP a cut-off of 3 SD was used.

### Specificity of HA1-specific indirect ELISAs

**Figure 2:**
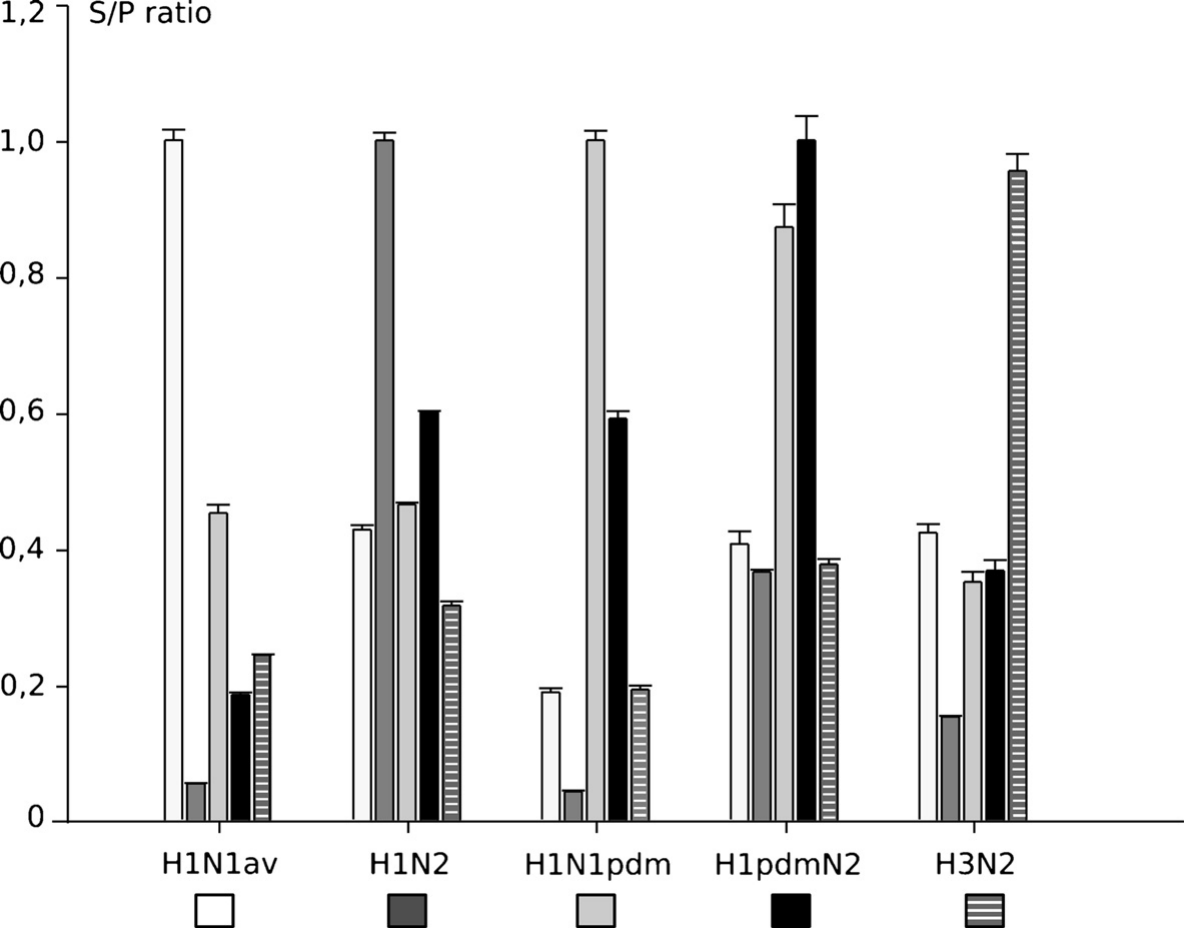
**Specificity of an indirect ELISA based on recombinant hemagglutinin fragment HA1 of porcine influenza A viruses.** Sera were obtained at day 21 of infection experiments in swine using the following viruses for inoculation: H1N1av – Porcine serum OM8 against A/swine/Belzig/2/2001, H1N2 – Porcine serum OM1 against A/swine/Bakum/1832/2000, H1N1pdm – Porcine serum OM46 against A/Bayern/74/2009, H1pdmN2 – Porcine serum OM15/4 against A/swine/Germany/R2035/2011, H3N2 – Porcine serum R4 against A/swine/Belzig/54/2001. The recombinant HA1 antigens were produced from the following viruses: H1N1av – A/swine/Germany/R1738/2010; H1N2 – A/swine/Germany/R1207/2010; H1N1pdm – A/Germany/R26/2011; H1pdmN2 – A/swine/Germany/R2035/2011; H3N2 – A/swine/Germany/96/2011.

Subsequently recombinant HA1-antigens were used to test sera obtained from experimentally infected pigs at day 21 after inoculation (Figure 2). HI assays confirmed a high specificity for these sera but also revealed minor cross reactivities especially between sera raised against subtype H1 viruses (Table 2). This was reflected in indirect ELISA in which the highest S/P ratios (set to 1.0) were consistently obtained with the homologous serum-recombinant HA1 protein pair. However, cross reactions were also evident by indirect ELISA, particularly among proteins of the H1 lineages. In general, cross reactions resulted in S/P ratios well below 0.5 although a serum raised against pandemic H1 could not distinguish pandemic H1 from reassorted H1pdmN2 and vice versa.

In order to test whether the observed serum cross reactivities were due to low-affinity antibody species, avidity assays were performed. Indirect ELISA assays were extended by an urea washing step for 10 minutes at room temperature before second antibody conjugate was added. As shown in Table 4, urea treatment diminished antibody reactivity against heterologous recombinant HA1 but had a significant negative influence on homologous HA1-serum reactions as well. Thus, urea treatment grossly decreased sensitivity with no apparent benefit on specificity. In further assays therefore urea washing steps were omitted.

**Table 4:** Serum avidity in indirect HA1 ELISA

**Recombinant HA**	**Serum specificity**								
	**H1N1pdm–A/Bayern/74/2009**^**a**^			**H1N1av –A/swine/Belzig/2/2001**			**H3N2 – A/swine Belzig/54/2001**		
	**No urea**	**Urea**	**AVI (%)**	**No urea**	**Urea**	**AVI (%)**	**No urea**	**Urea**	**AVI (%)**
A/Germany/R26/2011 (H1N1pdm)	**2,332**^**b**^	**1,273**	55	1,011	0,163	16	0,286	0,108	38
A/Swine/Germany/R2035/2011 (H1pdmN2)	1,359	0,266	20	0,292	0,109	37	0,306	0,099	32
A/Swine/Germany/R1738/2010 (H1N1av)	0,209	0,112	54	**1,241**	**0,312**	25	0,291	0,107	37
A/swine/Germany/R1207/2010 (H1N2)	0,176	0,16	91	0,078	0,061	78	0,087	0,06	69
A/swine/Germany/R76/2011 (H3N2)	0,182	0,111	61	0,159	0,121	76	**0,768**	**0,147**	19

^a^ Subtype and designation of influenza A virus isolate used to raise the respective serum.

^b^ Bold face indicates homologous rec HA1 – serum pair.

AVI (%) - percentage avidity index.

### Detection of influenza-specific antibodies in sera of experimentally infected swine

**Figure 3:**
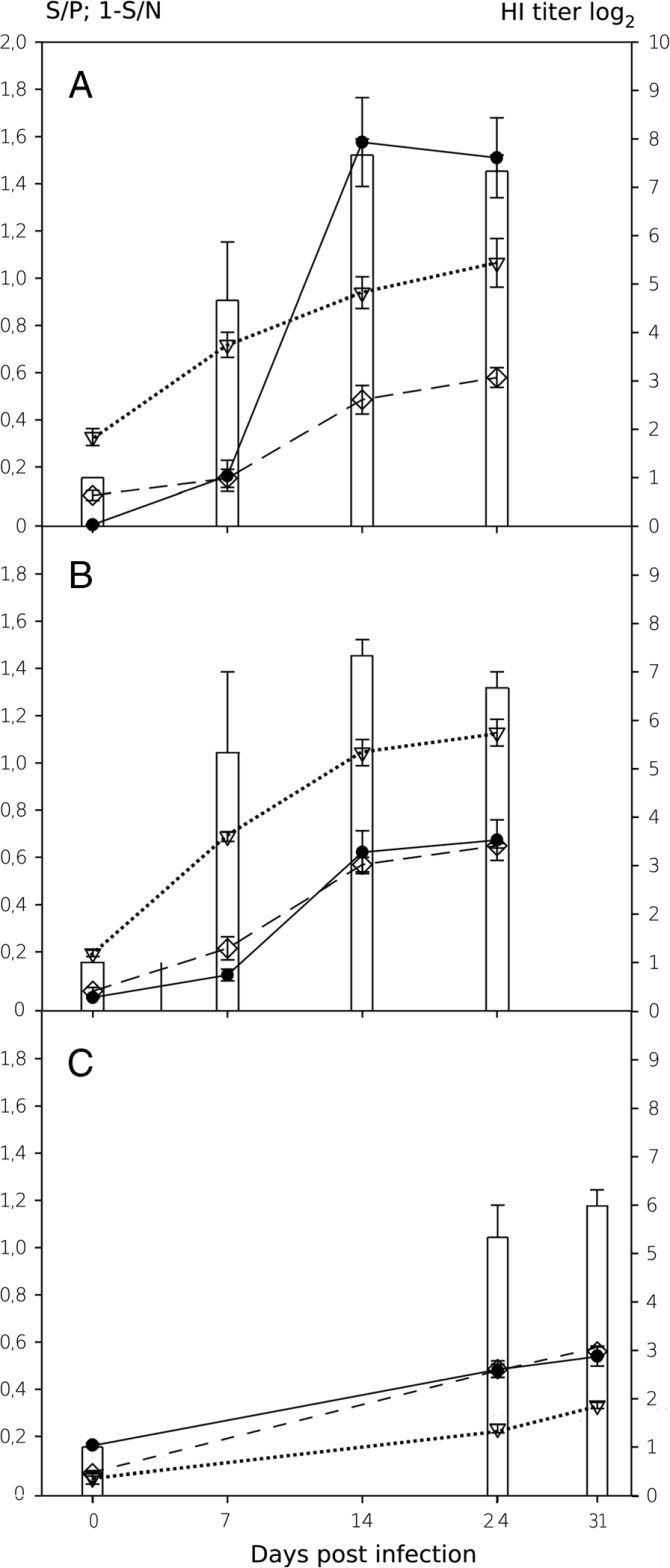
**Development of antibodies against viral nucleocapsid and hemagglutinin fragment HA1 proteins in swine experimentally infected with porcine influenza A viruses.** Experimental infection each of three pigs by **A** – A/sw/Germany/R708/2010 (H1pdmN1), **B** – A/sw/Germany/R2035/2011 (H1pdmN2), and **C** – A/sw/Germany/R248/2010 (H1N1av). Columns – HI titer against homologous virus antigen; open triangle – indirect recombinant NP ELISA; black dot – indirect recombinant HA1 ELISA (homologous antigen); open diamond – commercial blocking NP ELISA (ID.Vet). Samples of days 7 and 14 were unfortunately not available for experiment “C”.

In a next step the dynamics of antibody development after experimental influenza virus infections of pigs was examined. Experimental infections had been carried out previously using a pandemic H1 virus (A/sw/Germany/R708/2010), an H1pdmN2 reassortant (A/sw/Germany/R2035/2011), and an H1N1av isolate (A/sw/Germany/R248/2010). Sera obtained at different time points post infection were available for testing by a commercial NP blocking ELISA (ID.Vet, France), the indirect ELISAs specific for recombinant NP and for the homologous HA1 proteins, respectively. In addition, HI titers measured against the homologous virus were compared. Results are presented in composite Figure 3. The data show that antibody dynamics in experimentally infected swine can be accurately followed using the indirect ELISA formats. The results compare favorably with the commercial NP blocking ELISA and homologous HI.

### Detection of influenza-specific antibodies in porcine field sera

**Figure 4:**
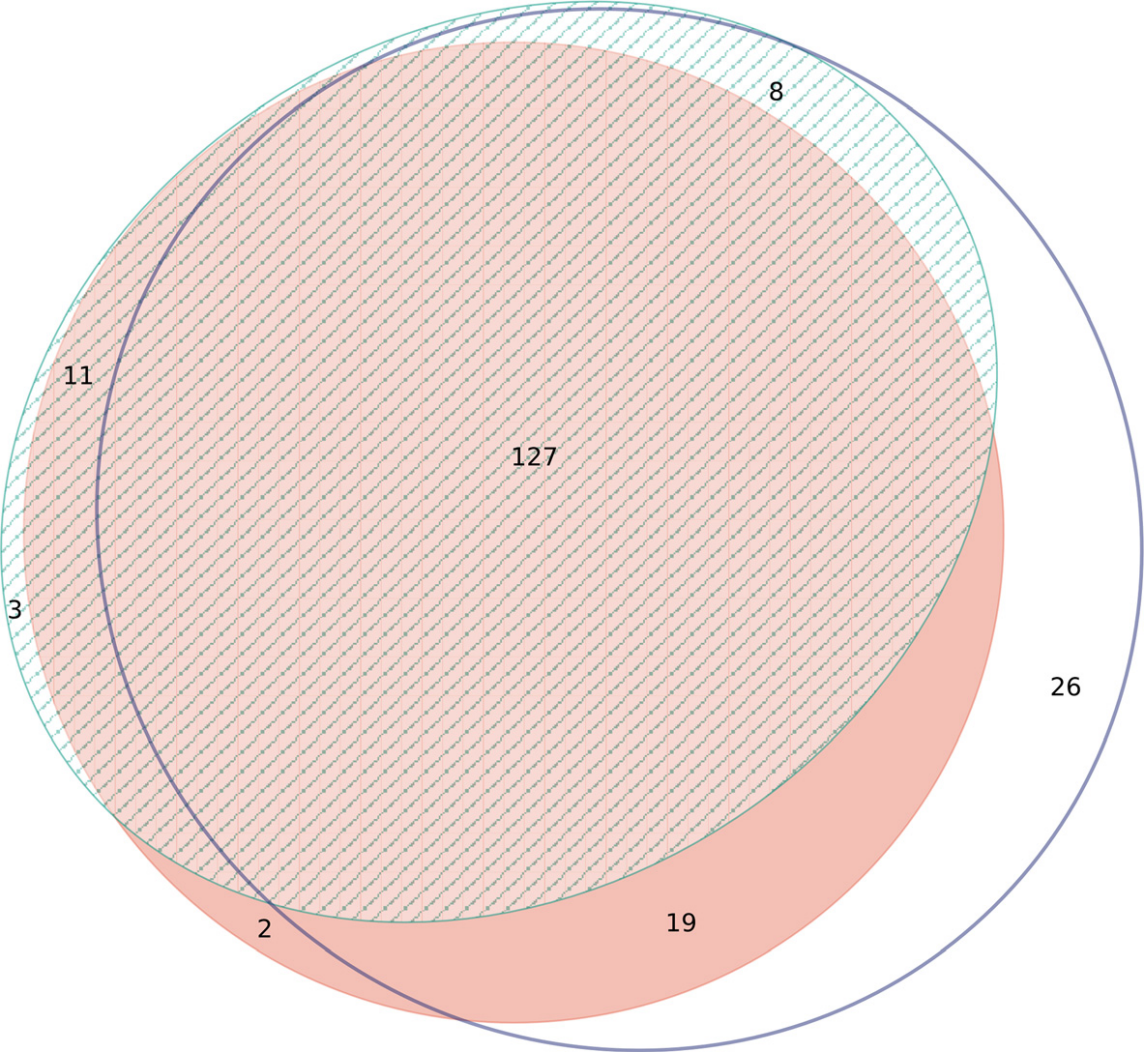
**Venn diagram of detection of NP-specific antibodies by commercial blocking ELISA (hatched ellipsis) and indirect ELISA (recombinant NP; red ellipsis) in porcine field sera (n = 207) compared with hemagglutination inhibition assays (white ellipsis).** Eleven sera produced congruently negative results in all three tests.

Finally, reactivity of a collection of 207 porcine field sera was compared by testing sera in HI assays, commercial NP blocking ELISA and indirect ELISAs with recombinant NP and HA1- antigens. These sera represented daily routine diagnostic submissions. As regards the HI assay, a serum was rated qualitatively positive for influenza A specific antibodies if it showed an HI titre of ≥ 40 (≥ 3 log_2_, starting dilution 1:10) against at least one of the four antigens (H1N1av, H1N2, H1N1pdm, H3N2) used for testing. Depicting the results of the commercial NP blocking ELISA, the indirect NP ELISA and the sum of the HI assays using a Venn diagram (Figure 4; [24]), an overall high degree of agreement was visually evident. The majority of results was backed by all three or at least two of the assays. However, in the HI assays, a comparatively high number of 26 sera revealed positive results just above the threshold (3 or 4 log_2_) which were not supported by other assays (Figure 4).

**Figure 5:**
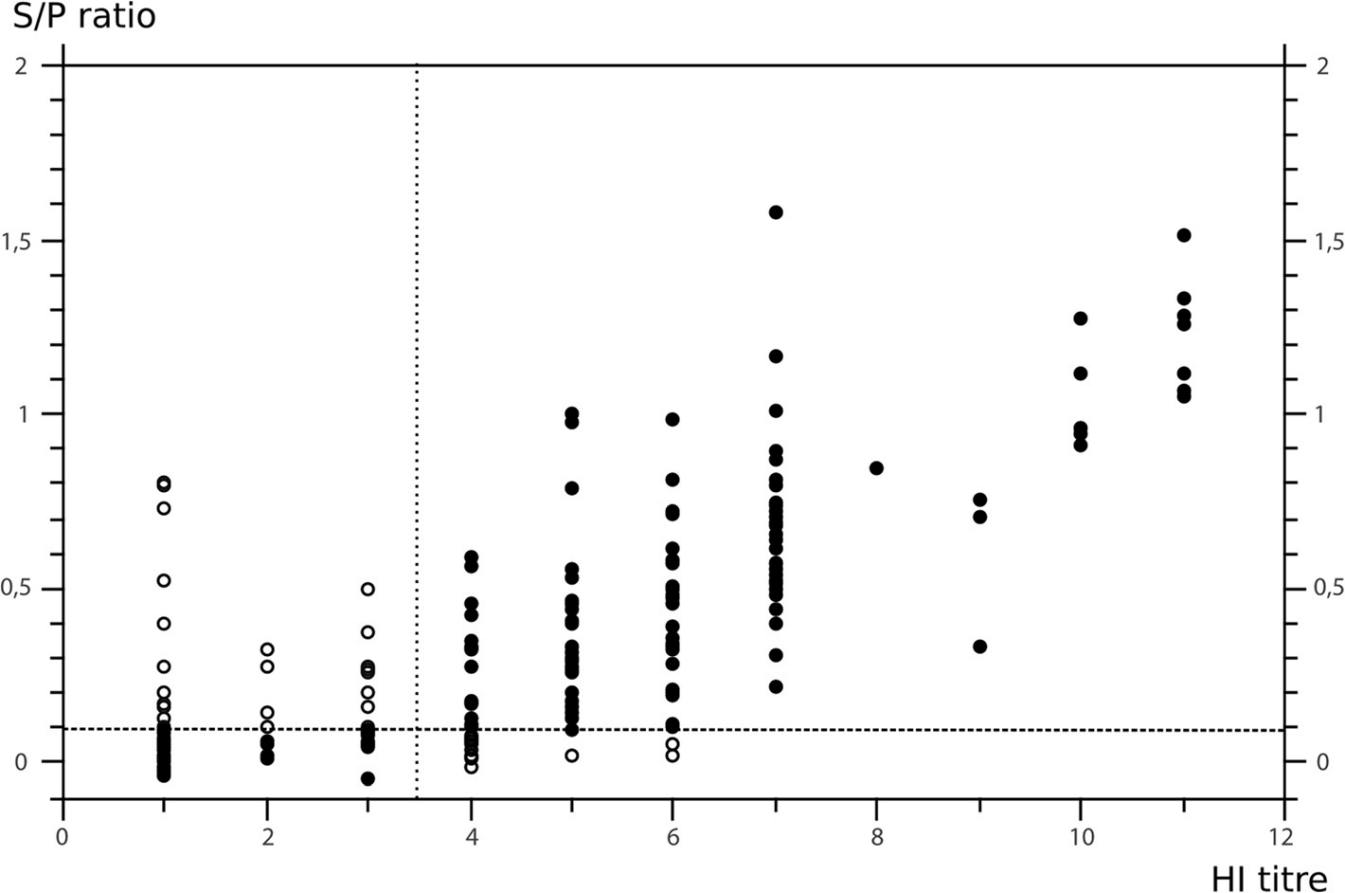
**Comparison of HI titres measured against porcine avian-derived H1N1 isolate A/swine/Germany/R248/2010 and S/P ratios obtained by indirect ELISA using recombinant HA1 antigen of avian-derived H1N1 isolate A/swine/Germany/R1738/2011.** Black dots – congruent qualitative result; white dots – incongruent qualitative result; HI titre shown as log_2_ series (starting dilution 1 = 1:10); dotted lines represent cut-off values.

Inter-rater agreement [25] was used to evaluate results of indirect ELISAs using recombinant HA1 compared to HI titres. While excellent and good agreements were seen for the H3 (κ_R96_ = 0,8255 [95% CI 0,6997-0,9513]) and the pandemic H1 antigens (κ_R26_ = 0,7772 [0,6917-0,8627]; κ_R2035_ = 0,661 [0,5643-0,7577]), respectively, only moderate agreement was signaled for the H1av (κ_R1738_ = 0,5722 [0,4401-0,7043]) and H1N2 (κ_R1207_ = 0,5083 [0,3359-0,6807]) antigens. A graphical analysis revealed that correlation between HI titres and S/P ratios was stronger for higher but less tight for sera with lower HI titres (Figure 5, examplified for recombinant H1av HA1 antigen). A good agreement (κ_HOLDING7_ = 0,625 [0,374-0,876]) across all indirect ELISAs was seen when results were compared based on holding level instead of individual sera (Table 5).

**Table 5:** Results of recombinant indirect ELISAs (iEIA) and HI or commercial NP-specific ELISA obtained with 207 porcine field sera compared on a holding base

**Holding**	**Sample**	**NP**		**H1av**		**H1N2**		**H1pdm**		**H1pdmN2**		**H3**	
		**cEIA IDVet**	**iEIA**	**HI**	**iEIA**	**HI**	**iEIA**	**HI**	**iEIA**	**HI**	**iEIA**	**HI**	**iEIA**
1	6^a^	5^b^	4	5	6	1	0	4	4	n.d.	2	0	0
2	16	15	12	14	16	3	0	13	16	n.d.	16	6	6
3	9	7	7	7	7	9	2	3	6	n.d.	4	5	3
4	7	7	7	7	7	7	7	7	7	n.d.	7	7	7
5	5	5	5	5	5	5	5	5	5	n.d.	5	5	5
6	30	30	28	30	29	1	0	28	26	n.d.	20	0	3
7	30	24	26	22	22	3	1	17	15	n.d.	14	0	0
8	30	13	18	8	14	0	0	1	3	n.d.	3	0	0
9	30	18	28	28	26	0	1	26	21	n.d.	15	0	0
10	23	3	12	9	7	3	0	0	0	n.d.	0	1	0
11	13	11	12	13	13	3	1	12	11	n.d.	6	0	0
12	10	10	10	10	6	1	1	5	3	n.d.	3	0	0

^a^ Number of sample obtained in holding.

^b^ Number of sample rated positive in the respective assay.

n.d. Not determined.

## Discussion

Serodiagnosis of porcine influenza virus infections in Europe and elsewhere is significantly challenged by co-circulation of several subtypes, and use of multivalent vaccines further complicates this situation. While generic antibodies directed against the well conserved influenza virus NP protein can be detected by commercial blocking ELISAs, the differentiation of subtype-specific antibodies requires use of the fastidious HI assay. Subtype-specific ELISA assays suitable for high throughput investigations of porcine sera would aid in promoting more intense studies on porcine influenza seroepidemiology.

Aiming to develop such assays we have successfully expressed recombinant full-length NP and HA1 fragments in bacteria and refolded proteins *in vitro*. Recombinant proteins were co-translationally mono-bitoinylated which facilitated purification and binding to solid, streptavidin-coated supports. Previous work by [26] has shown that bacterially expressed HA proteins can be refolded to acquire native conformation. Recombinant proteins representing recent isolates of the major subtypes and lineages of porcine influenza virus currently circulating in Germany were recognized by specific porcine or mustelid immune sera in Western blotting and indirect ELISA. Dynamics of generic and subtype-specific antibody development in experimentally infected swine as measured by recombinant indirect ELISAs fully paralleled results obtained by a commercial NP blocking ELISA and homologous HI assays.

However, despite use of the less conserved HA1 section of the HA glycoprotein as diagnostic antigen residual cross reactivity between the different subtypes was noticed in both Western blot (Figure 1) and indirect ELISA (Figure 2) even when using experimental post infection sera which were reasonably discriminatory between subtypes in HI assays (Table 2). Thus, a number of conserved epitopes exists between the different subtypes in the HA1 which is not detected by the functional HI assay. Yet, a considerable number of subtype-specific epitopes must have been represented in the recombinant HA1 proteins as well since, in indirect ELISA, the homologous HA1-serum pairs always resulted by far in the highest signal intensity (Figure 2). Human pandemic H1 HA1 represented by isolate R26/11 and that of the porcine-adapted variant H1pdmN2 R2035/11 were indistinguishable by Western blot and indirect ELISA although slight antigenic differences have been reported when using an HI assay [9].

The comparative examination of porcine field sera showed a variable agreement for the different recombinant antigens when individual sera were compared. In particular, ELISAs based on recombinant H1av and H1N2 revealed only moderate agreement when compared to HI. Fewer sera scored positive in the indirect ELISAs compared to HI assays. The majority of sera missed by the indirect ELISA showed low HI titres (3 or 4 log_2_) while reasonable correlation was seen between S/P ratios and HI titres for higher-titred sera (Figure 5). The blurring of results obtained with low-titred HI-positive sera may be due to low sensitivity of the indirect ELISAs but could as well have been caused by lack of specificity of the HI assays; problems with reproducibility and standardization of HI assays are notorious, especially when testing low-titred sera [20]. In addition, the HI assays here were carried out with antigens selected and used in routine diagnosis while the recombinant antigens were produced from more recent circulating viruses and differed slightly in HA1 amino acid sequences (not shown). This may have introduced further discrepancies as observed for individual sera. However, when results were compared on a herd basis a full match between HI and indirect ELISA results was evident (Table 5). Herds found to be seropositive by HI for a certain subtype were similarly positive in the respective HA1 indirect ELISA. Moreover, herds negative by HI for a certain subtype tested negative by the corresponding indirect ELISA. This indicates that on herd base the HI assay may be replaced, without loss of diagnostic quality, by the indirect ELISAs.

## Conclusions

Due to the ongoing antigenic diversification of porcine influenza viruses worldwide and new reassortant lineages springing up here and there serological diagnosis of porcine influenza becomes ever more demanding. Subtype and lineage-specific assays suitable for high throughput analysis will be required to cope with such diagnostic challenges. The recombinant mono-biotinylated HA1 proteins presented here as diagnostic antigens in indirect ELISAs provided an interesting alternative in this respect to HI assays. Further refinements of this strategy, e.g., by using lineage-specific monoclonal antibodies for competition or an immune-complex binding assay format, should be further investigated to replace HI assays.

## Material and methods

### Virus and cell culture

Influenza A viruses were propagated in serum-free MDCK cell cultures in the presence of TPCK-trypsin as detailed elsewhere [11]. Isolates were obtained from the virus repository maintained at the Friedrich-Loeffler-Institut. Molecular characteristics of recent porcine field isolates have previously been reported [9]. A list of viruses used in this study for production of recombinant proteins is provided in Table 1.

### Bacterial expression, *in vivo* biotinylation and purification of influenza virus HA1 and NP proteins

The HA1 fragments of the viral hemagglutinin open-reading frames (ORF) were cloned into the pET19b vector by a target-primed technique using Phusion polymerase amplification and *Dpn* I digested amplificates [27]. Sequences of primers are available on request. Expressed sequences stretched from the first amino acid of the mature protein to the arginin residue immediately proximal to the first glycin residue of the HA2 fusion peptide. Downstream of this arginin residue an Avi-Tag consensus sequence [28] was inserted. The central lysin residue of the 15 amino acid Avi-Tag sequence provides an acceptor site for covalent linkage of D-biotin which is specifically catalyzed by the bacterial biotin transferase *BirA*[29]*.*

HA1-pET19b expression constructs were co-transformed into Rosettagami *E. coli* with plasmid pBIRAcm (Avidity, Aurora, CO, U.S.A.) for overexpression of *BirA*. Dually transformed cells were selected using ampicillin and chloramphenicol (CM). Since CM is also required to maintain the genotype of Rosettagami *E. coli* cells, presence of both plasmids in selected colonies had to be confirmed by plasmid/insert-specific PCRs (primer sequences available on request). TYH medium supplemented with D-biotin at a concentration of 50 μg/ml was used for expression of co-translationally mono-biotinylatied Avi-tagged recombinant protein. The full length ORF of the nucleocapsid gene of the porcine influenza virus isolate R1738/10 was cloned and expressed similarly. However, the Avi-Tag was placed at the N-terminus of the protein. Lysates of Rosettagami cells transformed with plasmid pBIRAcm and an empty pET19b vector were used as a negative expression control.

Monobiotinylated bacterially expressed recombinant proteins were purified from inclusion bodies (IBs) by centrifugation and washing steps as previously described [26]. Proteins sequestered in purified IBs were then subjected to solubilization in 6 M guanidin-HCl and refolding using a panel of up to 30 different primary and up to nine secondary buffer conditions in a stepwise solubilization strategy using the ProteoStat kit (Enzo, Lörrach, Germany). Protein solubilization and reactivity were screened with conformation-dependent monoclonal antibodies and specific polyclonal sera in ELISA to sort out optimal refolding conditions for each of the recombinantly expressed proteins. Here, refolding conditions were used which had been validated using avian influenza virus H5 HA1 protein and two conformation-dependent monoclonal antibodies, 3H12 and 5 F3 (see [30], for properties of monoclonal antibodies). Final concentrations of recombinant proteins in appropriate refolding buffers were measured using a Coomassie protein assay kit (ThermoScientific, Rockford, IL, U.S.A.).

### Production of subtype-specific antisera

Pigs or ferrets were experimentally infected by the oronasal route with 10^6^ TCID_50_ of MDCK cell culture-grown influenza viruses in 1 ml cell culture medium using a nebulizer device (Wolfe Tory Medical, Salt Lake City, Utah) as previously described [31]. All experiments had received legal approval by an ethics commission (LALLF M-V/TSD/7221.3-2.5-004/10). Prior to infection animals were tested seronegative for influenza NP-specific antibodies in a commercial blocking ELISA (ID.Vet). Virus isolates used for infection are listed in Table 2. Blood samples used in further serological studies were obtained on day 21 post inoculation (p.i.). Reactivity of post infection sera in hemagglutination inhibition assays is detailed in Table 2.

### Origin of field sera

Porcine field sera from 12 swine holdings in Germany were submitted for routine diagnostic procedures. The history of these holdings for vaccination against influenza and/or clinical episodes of influenza virus infection was not documented.

### Indirect ELISA

Bacterially expressed proteins in refolding buffer were adjusted to a concentration of 5 μg/ml using TRIS-buffered saline (TBS). A total of 100 μl per well was used for binding to streptavidin-coated plates for 2 hours at room temperature or overnight at 4°C. Each plate included different recombinant antigens for each row with following strains: A:SIV/R1738/10 (H1N1),B: SIV/ R1207/11 (H1N2),C: –SIV/ R26/11 (H1N1pdm)D: SIV R2035/11 H1pdmN2, E: SIV/– R1931 /11 (H1N2), F: R76/11 (H3N2), G: R96/11 (H3N2), H: R1738/10 nucleocapsid protein. After washing wells were blocked using 5% nonfat milk-TBS containing 0.05% Tween 20 (TBST) (see Postel et al., 2011) for 2 hours at room temperature and then washed four times with TBST. Individual sera (100 μl per well, pre-diluted 1:200 in sample dilution buffer [ID.Vet, Montpellier, France]) were pipetted into columns (1A-1H) of the microtitre plate. This procedure assured that the reactivity of each serum against all antigens was measured in the same plate. Sera were incubated at room temperature for 1 hour. Wells were washed again four times with TBST before 100 μl of appropriately diluted goat-anti-swine IgG peroxidase conjugate (Dianova) was added for one hour at room temperature. Antibody was removed and after a final washing cycle with TBST, 50 μl of chromogenic TMB substrate was added. OD_450_ values were measured after 10 minutes of incubation and addition of 50 μl of 1 N H_2_SO_4_ to each well. Results were calculated and expressed in S/P units:

$$\frac{\mathrm{OD}\;\mathrm{Test}‒\mathrm{ODBackground}}{\mathrm{ODPositive}\;\mathrm{control}‒\mathrm{ODBackground}}\times 100=S/P$$

### Avidity measurement of sera by indirect ELISA

The indirect ELISA was performed as described above. However, after incubation of sera in the wells a washing step using urea in TBST was carried out. Different urea concentrations (0.5 and 2 M) and incubation times were evaluated, Final assays were carried out with 6 M urea for ten minutes at room temperature. Consecutive washing steps and conjugate incubation were carried out with TBST without urea. The sera were tested in parallel with and without the urea-buffer washing step and an avidity index (AVI) was calculated:

$$\frac{\mathrm{OD}\;\mathrm{sample}\;\mathrm{without}\;\mathrm{urea}}{\mathrm{OD}\;\mathrm{sample}\;\mathrm{with}\;\mathrm{urea}}\times 100=\mathrm{AVI}$$

### Generic nucleocapsid protein blocking ELISA (NP-bEIA)

For detection of group specific antibodies a commercial NP-bEIA was purchased (ID.Vet, Montpellier, France) and used according to recommendations of the manufacturer. Accordingly, samples were considered positive if the S/N (sample OD_450_/negative-control OD_450_ × 100) ratio was less than 45%, negative if the S/N ratio was more than 50%, and doubtful if the S/N ratio was between 45% and 50%.

### Hemagglutination inhibition assay (HI)

HI assays were performed according to O.I.E. recommendations essentially as described by [31]. Four hemagglutinating units of cell culture-grown influenza viruses were used throughout. All porcine and ferret sera were heat-inactivated for 30 minutes at 56°C and treated with receptor-destroying enzyme (neuraminidase from *Bacillus subtilis*).

## Abbreviations

AVI: Avidity index; IAV: Influenza A virus; H1av: Avian-derived porcine subtype H1; H1pdm: Human pandemic 2009-derived porcine subtype H1; HA: Hemagglutinin protein; HA1: Hemagglutinin fragment-1 protein; HI: Hemagglutination inhibition assay; NA: Neuraminidase protein; NP: Nucleocapsid protein; RT-qPCR: Real time reverse transcription polymerase chain reaction; TBS: TRIS-bufferdd saline; TBST: TRIS-buffered saline with Tween 20 supplement; TMB: 3,3′,5,5′-Tetramethylbenzidine.

## Competing interests

The authors declare that they have no competing interests.

## Authors’ contributions

ZN, CG and TCH conceived the study and drafted the manuscript. ZN carried out the molecular and serological work, and analysed samples. SK participated in the molecular and serological work. EL carried out animal experiments and provided samples. MB conceived the study, provided funds and edited the manuscript. All authors read and approved the final manuscript.
